# Study on risk factors of impaired fasting glucose and development of a prediction model based on Extreme Gradient Boosting algorithm

**DOI:** 10.3389/fendo.2024.1368225

**Published:** 2024-09-24

**Authors:** Qiyuan Cui, Jianhong Pu, Wei Li, Yun Zheng, Jiaxi Lin, Lu Liu, Peng Xue, Jinzhou Zhu, Mingqing He

**Affiliations:** ^1^ Department of Geriatrics, The First Affiliated Hospital of Soochow University, Suzhou, Jiangsu, China; ^2^ Physical Examination Center, The Affiliated Suzhou Hospital of Nanjing University Medical School, Suzhou, Jiangsu, China; ^3^ Department of Gastroenterology, The First Affiliated Hospital of Soochow University, Suzhou, Jiangsu, China; ^4^ Department of Endocrinology, The Affiliated Suzhou Hospital of Nanjing University Medical School, Suzhou, Jiangsu, China

**Keywords:** impaired fasting glucose, prediction model, artificial intelligence, cohort study, middle-aged and elderly people

## Abstract

**Objective:**

The aim of this study was to develop and validate a machine learning-based model to predict the development of impaired fasting glucose (IFG) in middle-aged and older elderly people over a 5-year period using data from a cohort study.

**Methods:**

This study was a retrospective cohort study. The study population was 1855 participants who underwent consecutive physical examinations at the First Affiliated Hospital of Soochow University between 2018 and 2022.The dataset included medical history, physical examination, and biochemical index test results. The cohort was randomly divided into a training dataset and a validation dataset in a ratio of 8:2. The machine learning algorithms used in this study include Extreme Gradient Boosting (XGBoost), Support Vector Machines (SVM), Naive Bayes, Decision Trees (DT), and traditional Logistic Regression (LR). Feature selection, parameter optimization, and model construction were performed in the training set, while the validation set was used to evaluate the predictive performance of the models. The performance of these models is evaluated by an area under the receiver operating characteristic (ROC) curves (AUC), calibration curves and decision curve analysis (DCA). To interpret the best-performing model, the Shapley Additive exPlanation (SHAP) Plots was used in this study.

**Results:**

The training/validation dataset consists of 1,855 individuals from the First Affiliated Hospital of Soochow University, yielded significant variables following selection by the Boruta algorithm and logistic multivariate regression analysis. These significant variables included systolic blood pressure (SBP), fatty liver, waist circumference (WC) and serum creatinine (Scr). The XGBoost model outperformed the other models, demonstrating an AUC of 0.7391 in the validation set.

**Conclusions:**

The XGBoost model was composed of SBP, fatty liver, WC and Scr may assist doctors with the early identification of IFG in middle-aged and elderly people.

## 1 Introduction

Type 2 diabetes mellitus (T2DM) represents a group of metabolic disorders marked by persistent elevations in blood glucose levels, posing a substantial global public health challenge. In 2017, it was estimated that 451 million individuals aged 18 to 99 years worldwide had diabetes, with projections indicating a staggering increase to 693 million by 2045, as reported by the International Diabetes Federation (IDF) (1). Prediabetes mellitus (PDM) signifies an intermediate phase preceding the onset of full-blown diabetes—a state of glucose metabolism lying between diabetes and normal glucose tolerance (NGT). It encompasses conditions like impaired fasting glucose (IFG), impaired glucose tolerance (IGT), or a combination of both. Recent data published in the British Medical Journal (BMJ) in 2020 by Chinese researchers revealed that the prevalence of diabetes among Chinese adults stood at 12.8%, with an alarming 35.2% prevalence in the prediabetic state (2). With the ongoing aging of society, the number of elderly individuals grappling with diabetes has surged dramatically. Notably, individuals with IFG face a significantly heightened risk of developing diabetes and its associated complications (3). The early identification of IFG in individuals and the timely implementation of lifestyle interventions can effectively mitigate the progression from IFG to T2DM (4).

Currently, relatively few studies have delved into risk prediction models specifically tailored to IFG, and many of these studies rely on cross-sectional data. For instance, a South Korean study (5) fashioned a predictive model for IFG using the categorical boosting (Cat Boost) algorithm, which encompassed eight predictors: age, high cholesterol levels, waist-to-hip ratio (WHtR), Body Mass Index (BMI), frequent alcohol consumption over the past year, marital status, hypertension, and smoking. Few investigations have concentrated on risk modeling for the development of prediabetes or IFG, as much of the existing literature primarily examines risk factors for IFG through cross-sectional analyses. For example, in a study by Khadija et al. that assessed prediabetes risk in nurses using straightforward statistical techniques, noteworthy variables associated with prediabetes included age, BMI, waist circumference (WC), antihypertensive medication history, high blood glucose history, family history of diabetes, daily consumption of fruits, berries, or vegetables, and daily physical activity (6). In summary, previous studies on risk factors for IFG are based on cross-sectional databases, which cannot provide causal associations for the development of IFG. In summary, it is proposed in this study to develop a predictive model for the development of IFG in middle-aged and elderly people using data from a longitudinal cohort study, which will provide valuable assistance to community healthcare providers and clinicians in the management of IFG.

In recent years, artificial intelligence technology has experienced rapid advancement, encompassing machine learning(ML), deep learning, and neural network algorithms (7). As such, they have found extensive utility in disease diagnosis and risk prediction within the medical and healthcare domains (8). ML algorithms encompass a range of techniques, including Extreme Gradient Boosting (XGBoost), Random Forest (RF), Support Vector Machines (SVM), Naïve Bayes, and more. These algorithms are distinguished by their capacity to learn from data, enabling them to make precise predictions regarding future events (9). In a study published in 2022, it was noted that an automated image analysis framework was constructed by using a simple convolutional neural network (CNN) model to recognize COVID-19 afflicted chest X-ray data. In order to improve classification accuracy, the fully connected layer of a simple CNN was further replaced with an efficient XGBoost classifier in the above study (10). The role played by ML algorithms can also be seen in non-medical fields, such as in India where academics have devised a Hierarchical Feature Selection (HFS) model based on Genetic Algorithms to optimize the local and global features extracted from each handwritten word images under consideration (11). In addition, there have been many advances in research related to Deep Learning (DL) algorithms, such as an enhanced version of the Firefly algorithm proposed in a study in 2021 that corrects the recognized shortcomings of the original method by explicitly exploring the mechanism and a chaotic local search strategy (12). In a proposed study published in 2022, an automated framework based on the hybridized sine cosine algorithm was proposed to tackle the overfitting shortcomings of neural network algorithms in DL algorithms (13). Consequently, the development of ML provides a novel avenue for constructing a predictive model for IFG. In the present study, risk factors for IFG were determined through a five-year longitudinal cohort analysis of clinical data. Considering DPN as an outcome variable, an accurate IFG risk prediction model was finally built based on multiple ML algorithms and traditional logistic regression (LR) analysis methods. Such an endeavor promises valuable insights into the prediction and prevention of IFG. In addition, this study is innovative in that it also uses a variety of visualization methods to demonstrate the role of weighting variables in the model output prediction results. This type of analytical approach to visualizing the implementation of ML algorithms has been less reported in risk prediction models for IFG. To improve the interpretability of the black-box model, the SHapley Additive exPlanation (SHAP) was used in this study to explain the predictive model. As a result, the prediction model not only predicts prognostic outcomes, but also provides reasonable explanations for the predicted outcomes, which greatly improves the user’s trust in the model. In summary, this study aims to establish a highly feasible model that provides a valuable reference for clinicians engaged in the early screening, diagnosis and treatment of IFG.

## 2 Methods and materials

### 2.1 Study population

The study population was selected from individuals who underwent health checkups at the Health Management Center of the First Affiliated Hospital of Soochow University. Data on health check-ups from January 2018 to December 2022 were collected for this study. To ensure the accuracy and reasonableness of the data, the research paid special attention to the last five years of data from the Health Management Center. According to the design of a retrospective cohort study, this study considered the 2018 health check-up data as the baseline of the cohort and the study ended in 2022. The presence of impaired fasting glucose (IFG) in this study population was the primary outcome of interest.

The inclusion criteria were as follows: (1) aged 45 years or older;(2) not previously diagnosed with IFG or T2DM at baseline;(3) possessed complete physical examination data from 2018 to 2022 without significant gaps in critical information. Exclusion criteria included: (1) age less than 45 years; (2) prior or recent diagnosis of IFG or T2DM at baseline; (3) use of medications that could influence plasma glucose levels;(4)missing or incomplete data on key clinical parameters such as fasting plasma glucose (FPG).

### 2.2 Data collection

**Figure 1:**
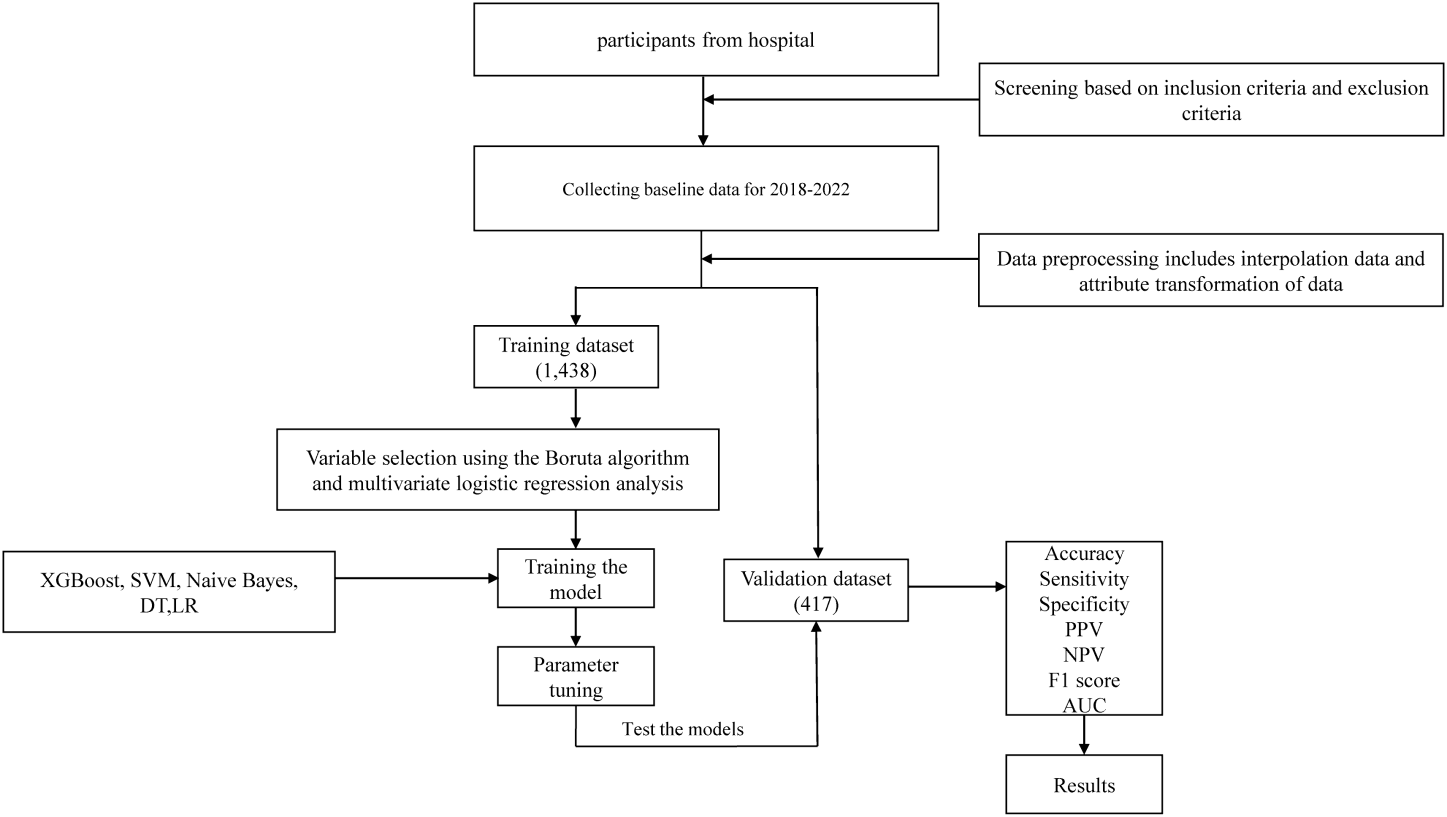
The flowchart of this study.

Demographic and sociological information, including age, gender, and medical history, was collected from all participants in this study. In addition, the results of each participant’s physical examination were recorded and laboratory measurements were taken. This involved measuring height, weight, systolic and diastolic blood pressure (SBP and DBP) and calculating the body mass index (BMI) based on the participant’s height and weight. All participants underwent fasting for a minimum of 8 hours before morning examinations, during which 3-5ml of venous blood was drawn from the elbow. Fasting plasma glucose (FPG), blood urea nitrogen (BUN), serum uric acid (SUA), serum creatinine (Scr), alanine aminotransferase (ALT), aspartic acid aminotransferase (AST), glutamyl transpeptidase (GGT), alkaline phosphatase (ALP), total cholesterol (TC), triglycerides (TG), high-density lipoprotein cholesterol (HDL), low-density lipoprotein cholesterol (LDL), apolipoprotein A1 (ApoA1), and apolipoprotein B (ApoB) were assessed using the Hitachi 7600 Automatic Biochemistry Analyzer. Furthermore, abdominal ultrasound examinations were performed by trained sonographers, and all participants underwent abdominal ultrasound scans. To accommodate machine learning algorithms that require numeric feature attributes, non-numeric attributes like gender were converted into numeric values. The percentage of missing variables included in this study was less than 30%. The MICE package was used for missing value analysis and multiple interpolation. **Supplementary Figure 1** illustrates the results of data interpolation. The flowchart of this study is shown in **Figure 1**.

### 2.3 Diagnostic criteria

In accordance with the World Health Organization (WHO) diagnostic criteria for diabetes mellitus established in 1999 (14), the FPG levels of the participants were classified in this study. A normal blood glucose state was characterized by a fasting glucose level below 6.10 mmol/L and a 2-hour glucose level from the oral glucose tolerance test (OGTT) under 7.80 mmol/L. IFG was defined as a fasting glucose level ranging from 6.10 to 7.00 mmol/L or a 2-hour OGTT glucose level below 7.80 mmol/L, not meeting the diagnostic criteria for diabetes. For the diagnosis of fatty liver, we adhered to the ultrasound criteria outlined in the National Workshop on Fatty Liver and Alcoholic Liver Disease, Chinese Society of Hepatology, Chinese Medical Association (15). In this study, the diagnosis of fatty liver was confirmed using ultrasound diagnostic criteria proposed at the National Symposium on Fatty Liver and Alcoholic Liver Disease of the Chinese Medical Association Hepatology Branch (15). Specifically, fatty liver diagnosis relied on one of the following criteria: (1) noticeable enhancement of near-field liver echoes surpassing that of the kidney; (2) indistinct intrahepatic duct structure; (3) gradual attenuation of liver echoes in the far field. Gallstones were identified through ultrasound imaging, typically presenting as one or more intense echoes within the gallbladder, extrahepatic bile duct, or intrahepatic bile duct, accompanied by movable acoustic shadows (16). A history of cholecystectomy denoted prior gallbladder removal, where the gallbladder was no longer visible on ultrasound (17). The term “gallstones” encompassed both the presence of gallstones and prior cholecystectomy (18).

### 2.4 Development of models

The data collected from the medical checkup center of the First Affiliated Hospital of Soochow University were randomly divided into a training set and a validation set at a ratio of 8:2. Feature selection, parameter tuning, and model building were performed in the training set, while the validation set was used to evaluate the predictive performance of the models. Variable selection was the main step before the modelling. Boruta algorithm belongs to one of the random forest algorithms, its main purpose is to screen and sort important characteristic variables related to dependent variables. In each iteration, a comparison of the importance of the original and shadow variables is performed. If the importance of the original variable is significantly higher than the importance of the shadow variable, the original variable is considered important; if the importance of the original variable is significantly lower than the importance of the shadow variable, the original variable is considered unimportant (19). Boruta algorithm reaches a specified limit of random forest operation after 99 iterations. All variables were selected by Boruta algorithm and then multivariate logistic regression analysis was performed. The filtered variables were incorporated into the ML model. ML prediction models were developed, comprising XGBoost, SVM, Naive Bayes, DT and LR models. To determine the optimal parameters, a 5-fold cross-validation grid search was executed on the training set, while the LR model was implemented with default parameters.

### 2.5 The evaluation and interpretation of models

Based on the prediction results of the model, the area under the receiver operating characteristic curve (AUC) of the training and validation sets can be calculated to assess the discriminative ability of the model. The confusion matrix, consisting of true positives (TP), true negatives (TF), false positives (FP), and false negatives (FN), was established to calculate Sensitivity, Specificity, positive predictive value (PPV), negative predictive value (NPV), accuracy (ACC), and F1 scores for evaluating discrimination performance of models. Formulas were as follows: Sensitivity=Recall= TP/(TP+FN); Specificity= TN/(TN+FP); ACC = (TP + TN)/(TP + FP + FN + TN); PPV = Precision = TP/(TP + NP); NPV = TN/(TN + FN); F1 score= 2*Recall*Precision/(Recall +Precision). Calibration curves plotted after sampling with repetition 500 times using the Bootstrap method reflect the fitting of the models. The decision curves (DCA) were used to assess the clinical utility of the predictive model. To gain insight into the factors that contribute to the development of IFG in middle-aged and elderly people, SHapley Additive Explanations (SHAP) plots were further developed in this study. These plots visually demonstrated the variable contributions to the outcome, with local SHAP plots providing a detailed look at variable contributions for specific instances. Feature importance was ranked according to the Shapley value. In addition, force plots within the SHAP model were used to individualize predictions for two randomly selected samples from the validation set.

### 2.6 Statistical analysis

Statistical analysis was performed using SPSS (version 27.0) to describe the general data of all individuals. The Shapiro–Wilk test was employed to ascertain the normal distribution of variables. Continuous variables displaying a normal distribution were presented as mean ± standard deviation (SD), while those with skewed distribution were described as median (interquartile ranges). Categorical variables were represented as frequencies. To compare variables between groups, the Pearson Chi-square test was used for categorical variables, while the Student’s t-test or the nonparametric Mann-Whitney U test was applied for continuous variables. All variables, determined as significant and tentative through Boruta algorithm screening, were incorporated into the multivariate logistic regression analysis. The logistic regression analysis was conducted using Enter approach, with α in = 0.05, α out = 0.1, and an inspection level of α = 0.05. SPSS (version 27.0) was also used to draw box plots to depict the independent risk factors associated with the onset of IFG in the middle-aged and elderly people identified in this study. The data interpolation, feature selection, model construction, evaluation, and visualization were executed using R software (version 4.2.3). The main packages involved are “mice, Boruta, caret, xgboost, shap for xgboost, shapviz, Resource Selection, rms”. A two-sided *p* < 0.05 was considered statistically significant.

## 3 Results

### 3.1 Demographic and clinical characteristics

A total of 1,855 individuals were included in the cohort, with 734 cases (39.0%) of impaired fasting glucose (IFG) observed within the entire cohort. They were randomly split into training (n = 1,438) and validation (n = 417) sets in an 8:2 ratio. In the training dataset, 73.0% (1,050/1,438) were male, and 27.0% (388/1,438) were female. The median age was 56 years (IQR = 50–67 years) for the IFG group and 55 years (IQR = 49–65 years) for the non-IFG group. In the test dataset, IFG onset was more common among male patients, with median ages ranging from 60 to 71 years. Detailed demographic and clinical characteristics are presented in **Table 1**.

**Table 1:** Demographic and clinical characteristics of participants.

Variables	The training dataset(n=1,438)				The validation dataset(n=417)		
	Group	IFG(n=570)	Non-IFG(n=868)	*p*-value	IFG(n=164)	Non-IFG(n=253)	*p*-value
Age(years) (median [IQR])		56.00[50.00,67.00]	55.00[49.00,65.00]	0.003	63.00[60.00,71.00]	62.50[54.00,69.00]	0.003
Gender(%)	Male	412(72.3)	638(73.5)	0.610	118(72.0)	189(74.57)	0.533
	Female	158(27.7)	230(26.5)		46(28.0)	64(25.3)	
Fattyliver(%)	Yes	267(46.8)	237(27.3)	<0.001	68(41.5)	75(29.6)	0.013
	No	303(53.2)	631(72.7)		96(58.5)	178(70.4)	
Gallstone(%)	Yes	102(17.9)	125 (14.4)	0.090	138(84.1)	35 (13.8)	0.569
	No	468(82.1)	743(85.6)		26(15.9)	218(86.2)	
Cholecystectomy	Yes	61(10.7)	60(6.9)	0.011	13(7.9)	15(5.9)	0.428
	No	509(89.3)	808(93.1)		151(92.1)	238(94.1)	
SBP(mmHg) (median [IQR])		136.00[123.00,150.00]	128.00[116.00,139.00]	<0.001	144.00[128.00,157.50]	132.00[21.00,145.75]	<0.001
DBP(mmHg) (median [IQR])		81.00[73.00,87.00]	78.00[70.00,84.00]	<0.001	80.00[71.50,87.00]	77.00[70.25,85.75]	0.001
WC(cm) (median [IQR])		90.00[85.00,96.00]	87.00[80.00,93.00]	<0.001	92.00[84.00,96.00]	88.50[81.25,94.75]	0.002
BMI(kg/m²) (median [IQR])		25.25[23.51,27.03]	24.49[22.52,26.17]	<0.001	24.77[22.84,27.18]	24.29[22.31,26.22]	0.001
BUN(mmol/L) (median [IQR])		5.10[4.50,6.00]	5.00[4.30,5.90]	0.024	5.10[4.15,5.90]	5.10[4.30,6.20]	0.217
Scr(μmol/L) (median [IQR])		70.95[60.30,80.03]	72.60[61.83,81.78]	0.068	72.00[61.20,81.45]	75.05[62.13,83.40]	0.961
SUA (μmol/L) (median [IQR])		367.15[313.28,418.15]	349.15[289.43,414.40]	0.001	388.40,319.50,440.95]	351.00[292.00,392.98]	0.340
ALT(U/L) (median [IQR])		21.40[15.60,29.00]	18.60[14.00,25.00]	<0.001	19.00[14.35,27.00]	17.25[13.03,22.90]	0.060
AST (U/L) (median [IQR])		21.00[17.90,25.30]	20.50[17.70,23.78]	0.038	19.00[17.00,23.10]	20.00[17.00,23.18]	0.110
GGT (U/L) (median [IQR])		26.85[19.10,41.73]	23.05[15.80,35.30]	<0.001	29.40[18.00,41.65]	21.35[14.20,31.25]	0.056
ALP (U/L) (median [IQR])		64.85[54.50,77.80]	63.45[53.93,75.68]	0.140	68.00[58.00,82.50]	64.95[53.20,76.95]	0.280
TC (mmol/L) (median [IQR])		4.41[5.04,5.69]	4.99[4.42,5.18]	0.094	4.90[4.31,5.57]	5.00[4.42,5.49]	0.640
TG (mmol/L) (median [IQR])		1.59[1.14,2.15]	1.34[0.93,1.91]	<0.001	1.54[1.21,2.26]	1.30[1.00,1.91]	0.080
HDL(mmol/L) (median [IQR])		1.18[1.00,1.40]	1.22[1.05,1.48]	0.003	1.19[1.06,1.35]	1.27[1.06,1.50]	0.434
LDL (mmol/L) (median [IQR])		2.87[2.31,3.39]	2.85[2.36,3.29]	0.305	3.08[2.55,3.72]	2.95[2.44,3.57]	0.380
ApoA1(g/L) (median [IQR])		1.38[1.25,1.53]	1.37[1.25,1.51]	0.892	1.39[1.22,1.52]	1.39[1.27,1.50]	0.922
ApoB(g/L) (median [IQR])		0.98[0.83,1.14]	0.95[0.82,1.08]	0.001	0.95[0.77,1.08]	0.97[0.80,1.09]	0.047

### 3.2 Feature selection

**Figure 2:**
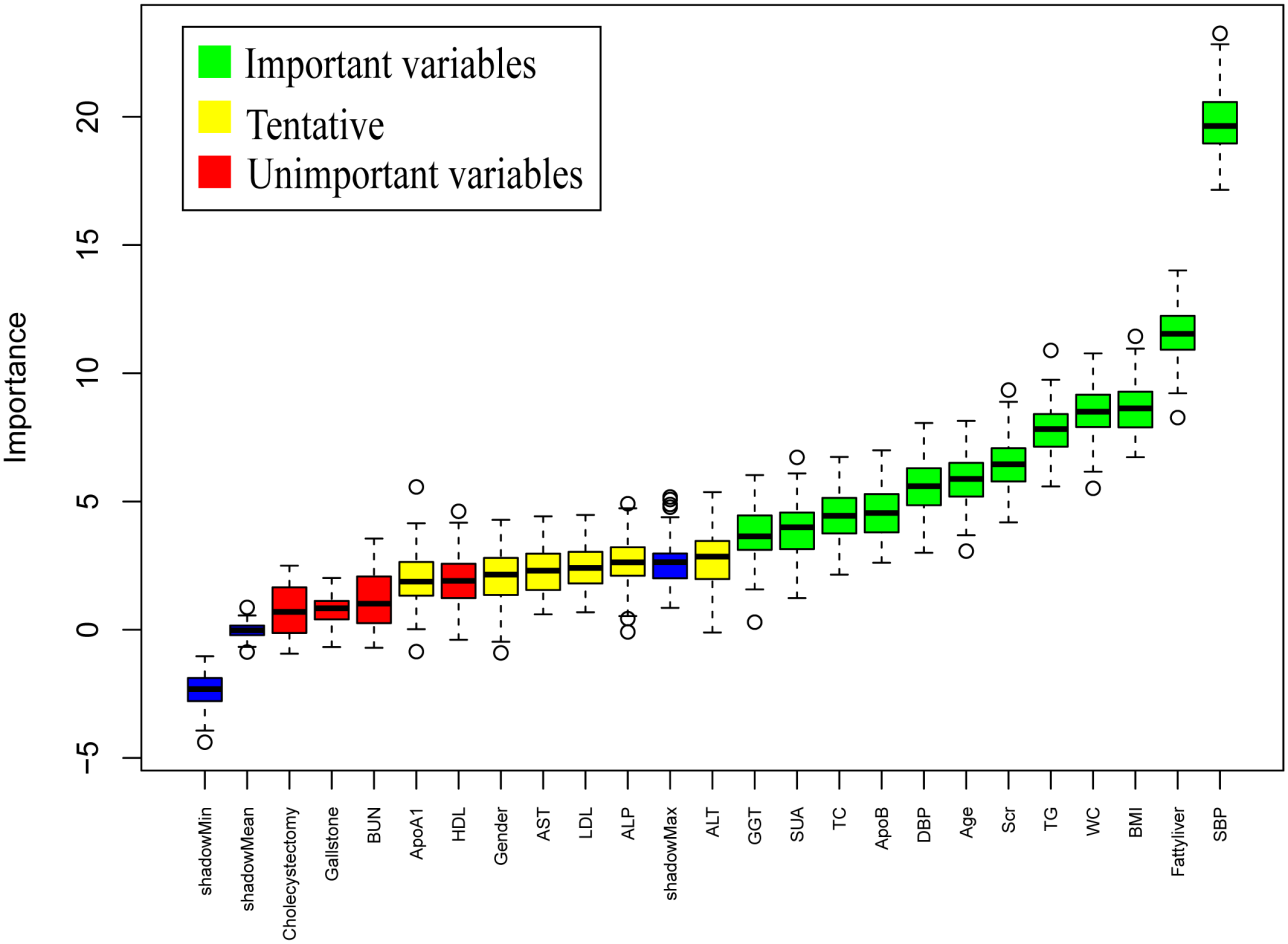
Results of variable selection by the Boruta method. SBP, systolic blood pressure; WC, waist circumstance; BMI, Body Mass Index; TG, triglycerides; Scr, serum creatinine; ALT, alanine aminotransferase; DBP, diastolic blood pressure; ApoB, apolipoprotein B; GGT, glutamyl transpeptidase; SUA, serum uric acid; AST, aspartic acid aminotransferase; TC, total cholesterol; LDL, low-density lipoprotein cholesterol; HDL, high-density lipoprotein cholesterol; BUN, blood urea nitrogen; ALP, alkaline phosphatase; ApoA1, apolipoprotein A1; Boruta method applied only to the training dataset.

**Figure 3:**
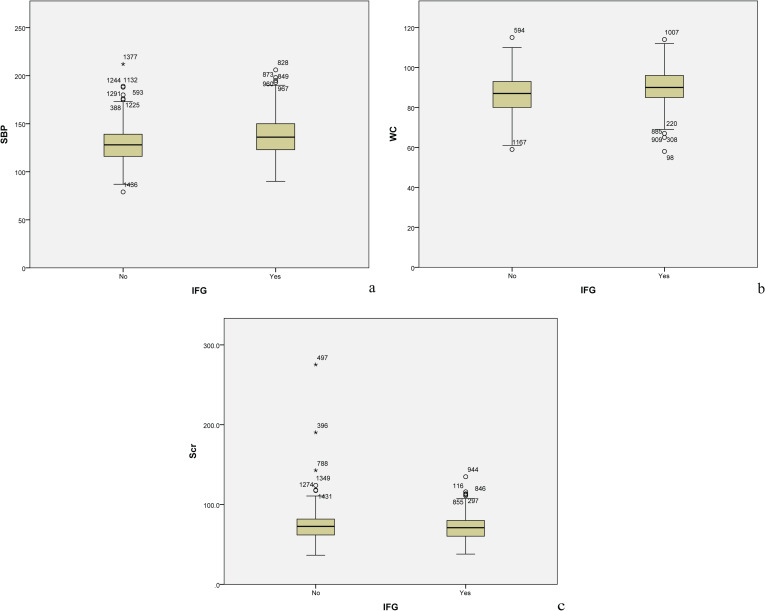
On the basis of the trainingset, box plots of the distribution of the three continuous variables in the IFG and non-IFG groups were further plotted. **(A)** This figure shows the box plot of the distribution of SBP in the IFG and non-IFG groups. **(B)** This figure shows the box plot of the distribution of WC in the IFG and non-IFG groups. **(C)** This figure shows the box plot of the distribution of Scr in the IFG and non-IFG groups.

Following 99 iterations, the Boruta algorithm’s feature variable screening results are illustrated in **Figure 2**. The following variables were considered important for their association with IFG: SBP, Fatty liver, BMI, WC, TG, Scr, Age, DBP, ApoB, TC, SUA and GGT. To further clarify the risk or protective factors related to IFG, multivariate regression analysis was conducted between the IFG and non-IFG groups in the training cohort, revealing significant differences in the following variables: SBP, Fatty liver, WC and Scr. This study confirmed that Scr was a protective factor associated with IFG, while SBP, Fatty liver and WC were all risk factors for IFG. The results showed that for every 1 mmHg increase in SBP, the risk of IFG in middle-aged and elderly people increased by 3.0% (OR =1.030, 95% CI: 1.020-1.040). The risk of IFG in middle-aged and elderly people increased by 3.3% for every 1 cm increase in waist circumference (OR = 1.034, 95% CI: 1.009-1.059). The risk of IFG was 50.5% higher in middle-aged and elderly people with fatty liver compared to those without fatty liver disease (OR = 1.657, 95% CI: 1.274-2.156). The risk of IFG in middle-aged and elderly people was elevated by 1.6% for every 1μmol/L decrease in Scr levels (OR = 0.984, 95% CI: 0.975-0.993). Further details are provided in **Table 2**. In the study, box plots describing the distribution of these three continuous variables including SBP, WC and Scr in the IFG and non-IFG groups were further plotted based on the training set. As shown in **Figure 3**, middle-aged and elderly people who developed IFG over a 5-year period had higher SBP, larger WC and lower Scr than middle-aged and elderly people without IFG.

**Table 2:** Multivariate logistic regression analysis of impaired fasting glucose.

Variables	β	Standard Error	Wald χ2	OR	95%CI		*p*-value
					Lower	Upper	
Fattyliver	0.505	0.134	14.147	1.657	1.274	2.156	<0.001
SBP	0.030	0.005	33.960	1.030	1.020	1.040	<0.001
WC	0.033	0.012	7.044	1.034	1.009	1.059	0.008
Scr	-0.016	0.005	11.533	0.984	0.975	0.993	0.001
Constant	-5.558	0.868	40.994	0.004			<0.001

### 3.3 The evaluation and interpretation of the models

**Figure 4:**
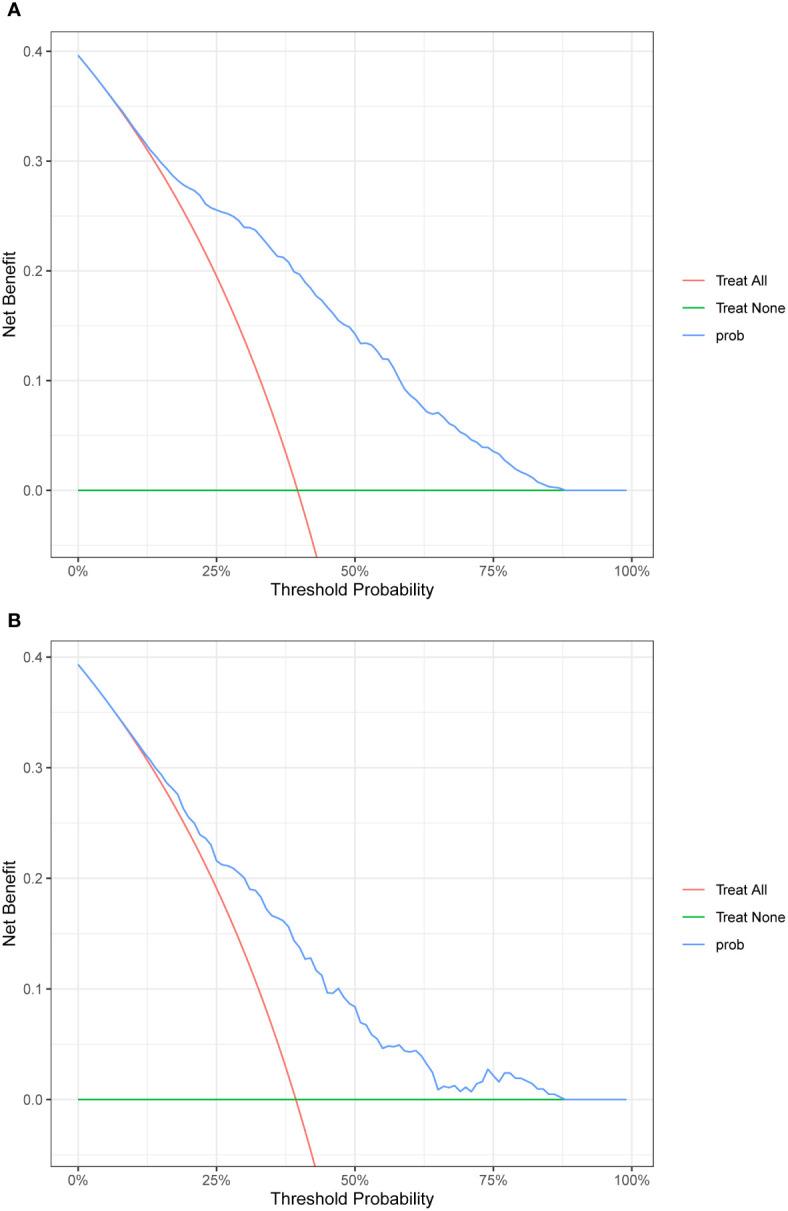
Calibration plot. The x‐axis represents the XGBoost model‐predicted probability, and the y-axis represents the actual probability of IFG. **(A)** The plot shows the calibration curve of the training set. A perfect prediction would fall along the 45‐degree line (“ideal” line). The “apparent” line represents the training cohort, and the solid black line represents bias corrected by bootstrapping (500 repetitions), indicating observed the performance of XGBoost model. **(B) **The plot shows the calibration curve of the validation set. A perfect prediction would fall along the 45degree line (“ideal” line). The “apparent” line represents the validation cohort, and the solid black line represents bias corrected by bootstrapping (500 repetitions), indicating observed the performance of XGBoost model.

**Figure 5:**
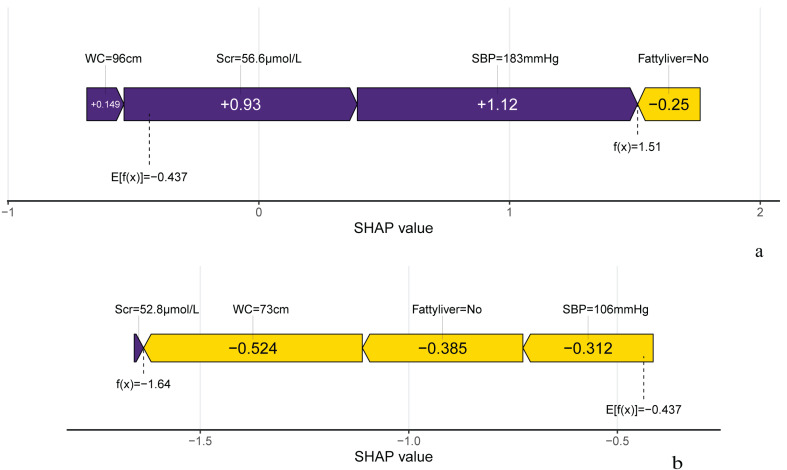
DCA plot. **(A)**The plot shows the calibration curve of the training set. **(B)**The plot shows the calibration curve of the validation set.

**Figure 6:**
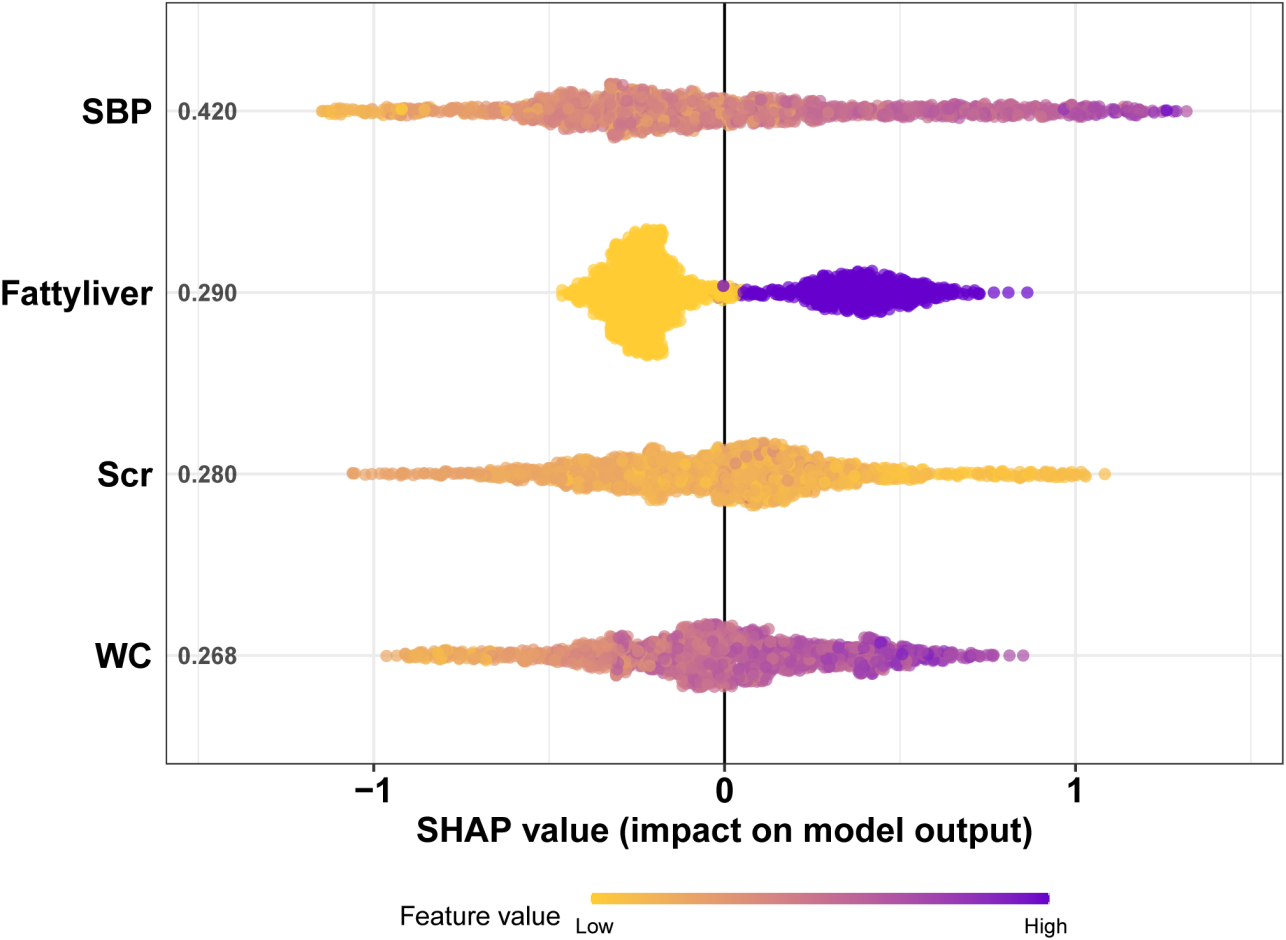
The SHAP plot of the XGBoost model. The ordinate represents the name of the variable. The variables from top to bottom are of decreasing importance to the predicted results and the number beside the variables is the mean of the SHAP values for all samples. Each point in the graph represents the SHAP value for each sample, with colors closer to purple indicating a larger value and closer to yellow indicating a smaller value. The more dispersed the points in the graph, the greater the influence of the variable on the model. **Figure 5** shows that SBP has the greatest impact on the model.

Four features selected by the Boruta algorithm and multivariate regression analysis were considered as input variables, with the development of IFG as the outcome. Six different algorithms, including XGBoost, SVM, Naïve Bayes, DT and LR, were applied to construct prediction models in this study. The AUC, Sensitivity, Specificity, ACC, PPV, NPV, and F1 scores of the model in the validation set were calculated from the confusion matrix results. The details are showed in **Table 3**. Among these models, the XGBoost model yielded the highest AUC, indicating superior performance. With the aid of grid search, the optimal structure of the XGBoost model was determined as follows: booster=‘gbtree’, objective=‘binary:logistic’,gamma=0.5, eta=0.06,max_depth=7, min_child_weight=5, subsample=0.65, colsample_bytree=0.72. **Table 3** displays the superior AUC value achieved by the XGBoost algorithm compared to SVM, Naive Bayes, DT and LR algorithms (0.7391, 0.7328, 0.7288, 0.6480 and 0.6795) respectively. Typically, a model with an AUC greater than 0.7 is considered to have good predictive performance. The calibration curves for the training and validation sets are plotted in this study and are shown in **Figure 4**. The results demonstrate the internal validation of the XGBoost model using the bootstrap method with 500 repetitions of sampling. The mean absolute errors for the training and validation sets were 0.010 and 0.025, respectively, indicating that the predicted probabilities of the XGBoost model closely aligned with the actual observations. The results of the Hosmer-Lemeshow goodness of fit test(H-L) showed that the model was well fitted (*p*>0.05). The clinical effect of the XGBoost prediction model was evaluated in this study using DCA curves, as shown in **Figure 5**, showing that individuals with a higher risk of developing IFG in middle-aged and elderly people as assessed using this XGBoost model may have a higher net benefit value if they were intervened. In addition, the SHAP framework has provided an intuitive interpretation of the XGBoost model, as shown in **Figure 6**.

**Table 3:** Predictive performance indicators of prediction models.

(a) Prediction performance of the training set.									
Model	AUC	95%CI		Sensitivity	Specificity	Accuracy	PPV	NPV	F1 score
		Lower	Upper						
**XGBoost**	0.8264	0.8079	0.8503	0.8561	0.6636	0.7399	0.6256	0.8754	0.7230
**SVM**	0.7230	0.7501	0.6959	0.2052	0.4516	0.3540	0.1973	0.4639	0.2012
**Naive Bayes**	0.6827	0.6548	0.7106	0.7673	0.4614	0.6460	0.5656	0.6845	0.7235
**DT**	0.6796	0.6557	0.7035	0.8318	0.4474	0.6794	0.6360	0.7030	0.7580
**LR**	0.6912	0.6637	0.7189	0.3947	0.8157	0.6488	0.5844	0.6724	0.4712

(b) Prediction performance of the validation set.									
Model	AUC	95%CI		Sensitivity	Specificity	Accuracy	PPV	NPV	F1 score
		Lower	Upper						
**XGBoost**	0.7391	0.6911	0.7870	0.7561	0.6245	0.6763	0.5662	0.7980	0.6475
**SVM**	0.7328	0.6838	0.7819	0.1646	0.4980	0.3669	0.1753	0.4791	0.1698
**Naive Bayes**	0.7288	0.6804	0.7773	0.7787	0.5549	0.6906	0.6190	0.7296	0.7533
**DT**	0.6480	0.6027	0.6932	0.8142	0.4817	0.6835	0.6270	0.7079	0.7235
**LR**	0.6795	0.6275	0.7316	0.3598	0.8735	0.6715	0.6484	0.6779	0.4627

AUC, Area Under the Curve; Extreme Gradient Boosting, XGBoost; DT, Decision Tree; SVM, Support Vector Machine; LR, Logistic Regression; PPV, Positive Predictive Value; NPV, Negative Predictive Value.

### 3.4 Individualized prediction of IFG

**Figure 7:**
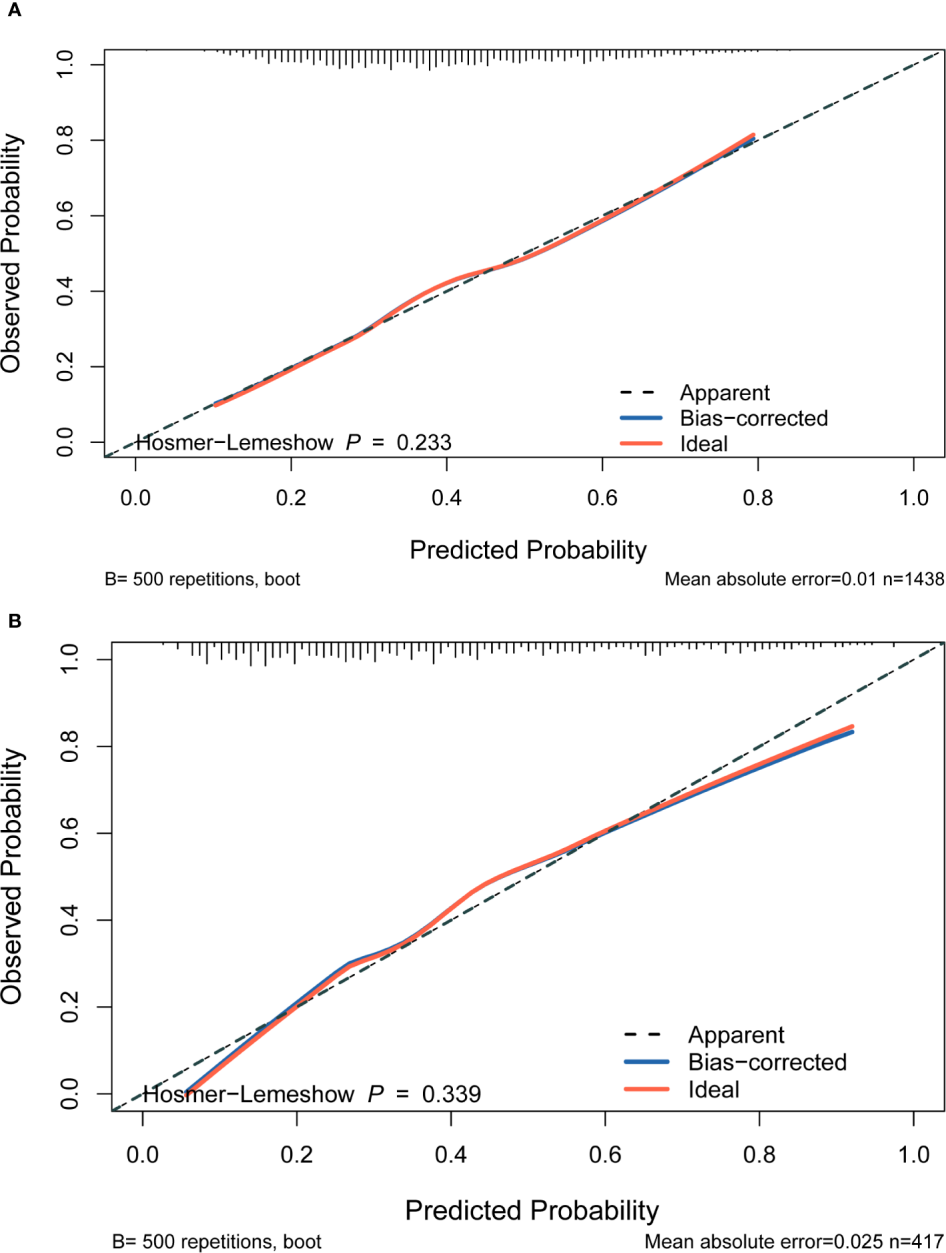
Predicted outcome of individual occurrence of IFG. **(A)** The local SHAP plot of the case#1. Case #1 Male, 72 years old, with a positive outcome at the end of follow-up and the model predicts an 81.9% probability of IFG in this study individual. **(B)** The local SHAP plot of the case#2. Case #2 Female, 54 years old, with a negative outcome at the finish of follow-up and the model predicts an 16.3% probability of IFG in this study individual.

**Figure 7** illustrates the SHAP analysis, showcasing the role of important variables on individual predictions in 2 randomly selected samples from the validation set. The local SHAP plots illustrated the contributions of variables to the outcomes for each sample. In **Figure 7**, the purple portion of the local SHAP force plot represents support for a positive prediction, while the yellow portion indicates support for a negative prediction. The length of the feature lines corresponds to the size of their contribution. For example, case 1 exhibited a high predicted probability of 0.82 for progressing to IFG, as predicted by the XGBoost model. SBP was the most significant feature contributing to the prediction, followed by Scr, Fatty liver and WC. This was enough to confirm the usefulness of the XGBoost model and contribute to increasing doctors’ trust in the predictive model to help them make the right auxiliary decisions.

## 4 Discussion

The strength of this study lies in the establishment of a retrospective cohort using clinical data from longitudinal physical examinations of middle-aged and elderly people from 2018 to 2022. The analysis of the original data can accurately reflect the real-world problems, and the results of the study have important reference value for further in-depth discussions. Furthermore, a prediction model for development of IFG was constructed by integrating the XGBoost algorithm. SHAP plots were used to visualize the model, which solved the limitations imposed by the “black box” nature of traditional machine learning. Boruta’s algorithm screened a total of twelve features deemed important in relation to IFG. To further clarify the risk or protective factors related to IFG, multivariate regression analysis was conducted between the IFG and non-IFG groups in the training cohort. Four features were ultimately selected for inclusion in the model and it was concluded that all of these variables are readily available in routine medical practice and therefore have clinical value. In clinical practice, the accurate identification or screening of IFG presents a crucial opportunity to control diabetes progression and reduce its associated harm.

Research has indicated that insulin sensitivity gradually decreases over time, a phenomenon that manifests a decade before the diagnosis of T2DM (20). This finding provides evidence that insulin resistance(IR) and the function of pancreatic β-cells are diminished when the body is in the stage of IFG (21). Hypertension is a common diabetes complication, with a study by Emdin et al. revealing that for every 20 mmHg increase in SBP, the risk of new-onset T2DM rises by approximately 58% (22). Nonlinear associations between blood pressure and diabetes risk have been observed, with SBP demonstrating a J-shaped curve, while DBP shows a U-shaped curve (23). Additionally, this study underscores the interaction effect of high SBP (>200 mmHg), low DBP (<69 mmHg), and age (>50 years) in increasing diabetes risk. SBP emerges as a more significant contributor to dysglycemia compared to DBP, particularly in individuals over 50 years old (24). These findings align with the present study, which suggest that elevated SBP has a greater influence on IFG occurrence than elevated DBP. WC reflects abdominal obesity, which, when present, leads to IR, characterized by impaired insulin response in peripheral tissues, and altered glucose uptake and utilization (25). Several studies, including this one, have found strong correlations between IFG and obesity-related indicators such as WC and BMI, underscoring their ability to shift glucose metabolism from normal to impaired in middle-aged and elderly people.

The SHAP algorithm, rooted in game theory, allows for the analysis of feature contributions to model predictions, offering both local and global interpretations. Each feature is considered a contributor, and its marginal contribution is calculated when added to the model (26). The SHAP model’s key advantage is its capacity to reflect the influence of features in each sample, including both positive and negative effects on predictions. SHAP analysis in this study demonstrates that lower Scr levels are associated with a higher likelihood of IFG. This aligns with previous research suggesting that low creatinine levels may correlate with IFG onset, even after adjusting for variables such as age, BMI, and SBP (27). Creatinine levels serve as a proxy for skeletal muscle mass, with lower levels indicating reduced muscle volume and fewer insulin targets (28). Thus, lower Scr levels may contribute to IFG (29). The results obtained by the model helped better understand the importance of each feature to the model’s prediction. Among the indicators detected by the model, the four most closely related to IFG were SBP, Fatty liver, WC and Scr. The high correlation between the above variables and IFG further emphasized the importance of early intervention in preventing development of IFG and T2DM.

There are some theoretical and practical limitations in this study. At present, deep learning algorithms can be used for both structured and unstructured data. In the future, unstructured data needs to be collected and combined with deep learning algorithms applied to the research field of risk prediction models for predicting the development of IFG. Secondly, in the practice of data collection, the number of participants in this study is small, so it is necessary to further expand the sample population and joint multi-center studies in the future. Potential factors contributing to IFG, such as dietary habits and lifestyle, were not considered in this study, and future studies should incorporate these variables with genetic information and nutritional intake for a more comprehensive understanding of IFG.

## 5 Conclusions

In conclusion, this cohort study developed a predictive model for IFG development using the XGBoost algorithm, demonstrating promising performance. This effective computer-assisted approach can aid frontline clinicians in recognizing and intervening in IFG development. Consequently, it advances the frontiers of T2DM prevention through more effective early identification and mitigation of the disease’s negative impact on middle-aged and elderly people.

## Data Availability

The raw data supporting the conclusions of this article will be made available by the authors, without undue reservation.
