# Supplementary material for: Study on risk factors of impaired fasting glucose and development of a prediction model based on Extreme Gradient Boosting algorithm
Source: Front Endocrinol (Lausanne). 2024 Sep 24;15:1368225. doi: 10.3389/fendo.2024.1368225 (PMC11458394; doi:10.3389/fendo.2024.1368225)
Supplement: Supplementary file 1 [file DataSheet1.docx]

Supplementary Material

**Study on risk factors of** **impaired fasting glucose and development of a prediction model based on Extreme Gradient Boosting (XGBoost) algorithm**

**Qiyuan Cui^1†^, Jianhong Pu^1†^, Wei Li^2†^, Yun Zheng^1^, Jiaxi Lin^3^, Lu Liu^3^, Peng Xue^4*^, Jinzhou Zhu ^3*^and Mingqing He^1*^**

*** Correspondence:**

Mingqing He, the First Affiliated Hospital of Soochow University, 188 Shizi Street, Suzhou, Jiangsu Province,215006, China. E-mail address: [hmqiori@163.com](mailto:hmqiori@163.com)

Jinzhou Zhu, the First Affiliated Hospital of Soochow University, 188 Shizi Street, Suzhou, Jiangsu Province,215006, China. E-mail address: [jzzhu@zju.edu.cn](mailto:jzzhu@zju.edu.cn)

Peng Xue, the Affiliated Suzhou Hospital of Nanjing University Medical School, 1 Lijiang Road, Suzhou, Jiangsu Province,215153, China. E-mail address:

xsytyl@126.com

# Supplementary Figures and Tables

## Supplementary Figures


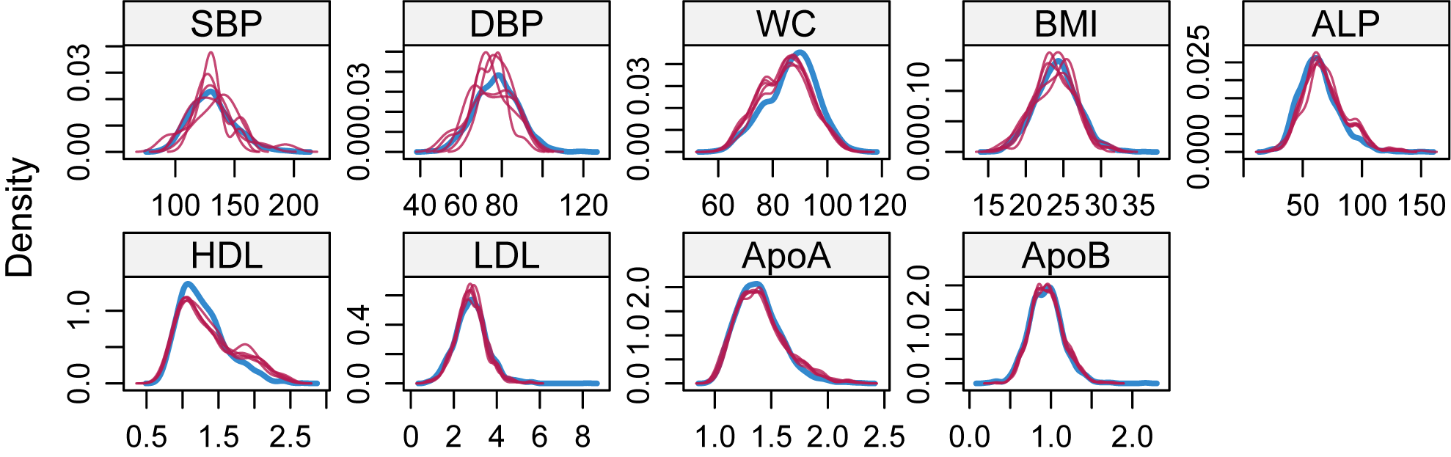


**Supplementary Figure 1.** The percentage of missing information was less than 30% for all the study subjects and data interpolation was done for less missing data. The missing data was interpolated using the "mice "package in R software, and the density plots were used to view the distribution of the interpolated data set with the original data.
